# Categorization of Extremely Brief Auditory Stimuli: Domain-Specific or Domain-General Processes?

**DOI:** 10.1371/journal.pone.0027024

**Published:** 2011-10-27

**Authors:** Emmanuel Bigand, Charles Delbé, Yannick Gérard, Barbara Tillmann

**Affiliations:** 1 Centre National de la Recherche Scientifique, UMR5022, Laboratoire d'Etude de l'Apprentissage et du Développement, Université de Bourgogne, Dijon, France; 2 Centre National de la Recherche Scientifique, UMR5292, Institut National de la Santé et de la Recherche Médicale, U1028, Lyon Neuroscience Research Center, Auditory Cognition and Psychoacoustics Team, Lyon, France; 3 University of Lyon, Lyon, France; 4 University of Lyon, Villeurbanne, France; Nothwestern University, United States of America

## Abstract

The present study investigated the minimum amount of auditory stimulation that allows differentiation of spoken voices, instrumental music, and environmental sounds. Three new findings were reported. 1) All stimuli were categorized above chance level with 50 ms-segments. 2) When a peak-level normalization was applied, music and voices started to be accurately categorized with 20 ms-segments. When the root-mean-square (RMS) energy of the stimuli was equalized, voice stimuli were better recognized than music and environmental sounds. 3) Further psychoacoustical analyses suggest that the categorization of extremely brief auditory stimuli depends on the variability of their spectral envelope in the used set. These last two findings challenge the interpretation of the voice superiority effect reported in previously published studies and propose a more parsimonious interpretation in terms of an emerging property of auditory categorization processes.

## Introduction

Event recognition in everyday life can be triggered by very brief stimuli. In the auditory modality, Grosjean's gating experiments demonstrated that spoken words can be recognized after 240 ms [1]. In this paradigm, participants were presented with voice stimuli of increasing duration, which they have to recognize. Effects of voice familiarity [2], voice gender [3], human *versus* computer voice [4], voice repetition priming [5], speech *versus* musical tones [6], speaker identity [7] and voice expression [8] (also see [9]) have been observed for stimuli with durations between 150 ms to 200 ms. Fast processing for perceiver-relevant stimuli, such as faces or voices, was claimed to rest on highly specialized pathways ([10], [11] for faces; [12]–[17] for voices).

Evidence for an advantage of voice processing was also reported with ERP measurements. Brain responses to vocalizations (mostly human) were distinguishable from responses to sounds from man-made auditory objects 70 ms after stimulus onset [18]. In this last study, musical sounds were part of the category of man-made objects, and they had not been analyzed separately. In another study, human vocalizations (73 speech items, 77 vocalizations), and other every-day life sounds (30 natural sounds, 60 instruments and 60 mechanical sounds) led to different ERP responses [19]. It was reported that as early as 164 ms post-stimulus-onset, the amplitudes of ERPs at fronto-temporal electrodes were consistently larger for voices than for bird songs and environmental sounds. At 200 ms, the electrophysiological response to voices reached nearly twice the amplitude of the ERPs to the other sound types. These findings have provided evidence for an early electrophysiological response to human voices, referred to as the “fronto-temporal positivity to voices” (FTPV), which is comparable to the well-known face preferential N170. Note that in this study [19], instrumental sounds were also included in the large category of environmental sounds and were not directly compared with vocalizations. Finally, sung voices and musical instrument sounds (e.g., violin, alto, cello, and brass) were found to be distinguishable from each other in ERP responses 320 ms after stimulus onset, notably in a fronto-central distribution [20]. This component was called the “voice-specific response”.

Up to now, specialized pathways for environmental sounds have not been identified yet, but some authors have suggested that music might be processed by dedicated modules [21], [22]. Some aspects of music processing were actually found to occur in an extremely fast and automatic way. The most astonishing finding was that musical excerpts of a duration of 100 ms allowed the identification of five famous pop tunes [23]. When an open set of tunes is used, participants can differentiate familiar versus unfamiliar music for excerpts as short as 500 ms [24], [25], and 100 ms [26]. To the best of our knowledge, only one single behavioral study has compared the temporal dynamics of music and voice processing [27]. Sung vowels and musical instrument sounds (bassoon, clarinet, oboe, piano, saxophone, and percussion) were used, in both reaction time and gating experiments. In a “go/no go” recognition task, responses to sung vowels as target stimuli were faster than responses to percussion and strings as targets. However, as other instrumental sounds (i.e., bassoon, oboe, and saxophone) were used as distractor items, this finding did not necessarily demonstrate a voice-processing advantage. A more parsimonious account suggests that the acoustic distances between target and distractors were stronger when sung voices defined the targets than when percussion or strings defined the targets. In addition, it remains difficult to generalize the conclusion of this study to everyday-life sounds. Sung vowels are weakly representative of spoken voice, and isolated musical sounds (e.g., tones) are weakly representative of music.

All of these studies lead to the conclusion that auditory categorization can be achieved on the basis of very little information, notably for voices. These findings may be understood within two different theoretical frameworks. According to a modular approach, the processing of voice is domain-specific and rests on highly specialized pathways [12]–[17], [27]. A modular process acts in a fast and automatic way [28], which leads to an advantage of voice processing. An alternative framework proposes that auditory stimuli are processed by general perceptual categorization mechanisms that rest on the analysis of the distribution of their perceptual features (e.g., [29], [30]; see also [31], [32] for a similar proposal in the visual domain). Within this framework, a processing advantage for a given class of sounds may be the consequence of the distances and the variability of the perceptual features of the exemplars used in the experimental setting.

Our present study used a gating procedure to further assess the specificity of the processes that govern auditory categorization. Compared to previous studies, we compared three classes of everyday-life sounds (voices, musical sounds, and environmental sounds). The goal was to identify the minimum duration allowing the differentiation of these categories of sounds. Moreover, we investigated the effect of two types of amplitude normalization (peak-level and RMS normalization). Studies reporting a voice superiority effect [27], as well as those investigating the neural sensitivity to human voices (e.g., [14], [19], [20]) used the RMS normalization. Up to now, no study has compared the RMS normalization with other normalization types, such as the Peak-level normalization, which is another commonly used normalization procedure. Comparing two normalization procedures has implications for the two alternative frameworks presented above (i.e., modular versus general perceptual categorization). A modular approach of sound processing could hardly predict an effect of amplitude normalization. By contrast, a general categorization process could anticipate such an effect by considering how it modulates the distance and distribution of perceptual features within and between sound categories (here, the exemplars used in the experimental setting).

## Materials and Methods

### Participants

Thirty-seven first-year psychology students of the University of Bourgogne participated in the experiment. They were 18 to 25 years old, none reported any auditory deficit, and none had formal training in music. Eighteen participants were assigned to the Peak-normalization condition, and 19 participants to the RMS-normalization condition.

### Ethics Statement

The study was performed in a pedagogical context, in which students, in exchange for course credits, have to participate in a non-invasive laboratory experiment, to further their understanding of experimental psychology. Informed written consent was obtained from all participants prior to taking part in the experiment. The study was anonymous and fully obeyed to the Helsinki Declaration, Convention of the Council of Europe on Human Rights and Biomedicine.

### Stimuli

Auditory samples of 30 s duration were selected in the following way. Samples of instrumental classical music belonging to the classic and romantic symphonic repertoires (see Table S1) were selected. As classical and romantic symphonic music are sub-categories of music, a similar sub-categorization was applied to the samples of human voices. Single spoken voices of man and female speakers were recorded from FM French radio. A subset of environmental sounds (referred to as ESounds here below) was selected from various audio CDs. All these sounds had high probability of occurrence in our everyday-life environment (see [33]). Peak normalization was applied to all auditory samples before being segmented in short excerpts (defining the “Peak-normalization” condition). This segmentation was performed by an home-made algorithm implemented in Matlab, which randomly selected experimental excerpts with durations of 20 ms, 30 ms, 50 ms, 100 ms and 200 ms. Silent or quasi silent excerpts were removed. In the “RMS-normalization” condition, the short excerpts further received a Root Mean Square (RMS) normalization for amplitude. Twenty excerpts for each category of sounds (3) were used in each duration condition (5), generating 300 (20×3×5) stimuli. Each participant heard the 300 stimuli played through SENNHEISER headphones.

### Procedure

Participants were invited to classify the stimuli in three main categories of sounds: Human voice, musical and environmental sounds (ESounds). The experiment was performed by blocks, starting by presenting all stimuli of 20 ms (in a random order), then continuing without pause with all stimuli of 30 ms and so on up to the 200 ms stimuli. There was neither a training phase nor response feedback.

## Results

### Behavioral data

**Figure 1:**
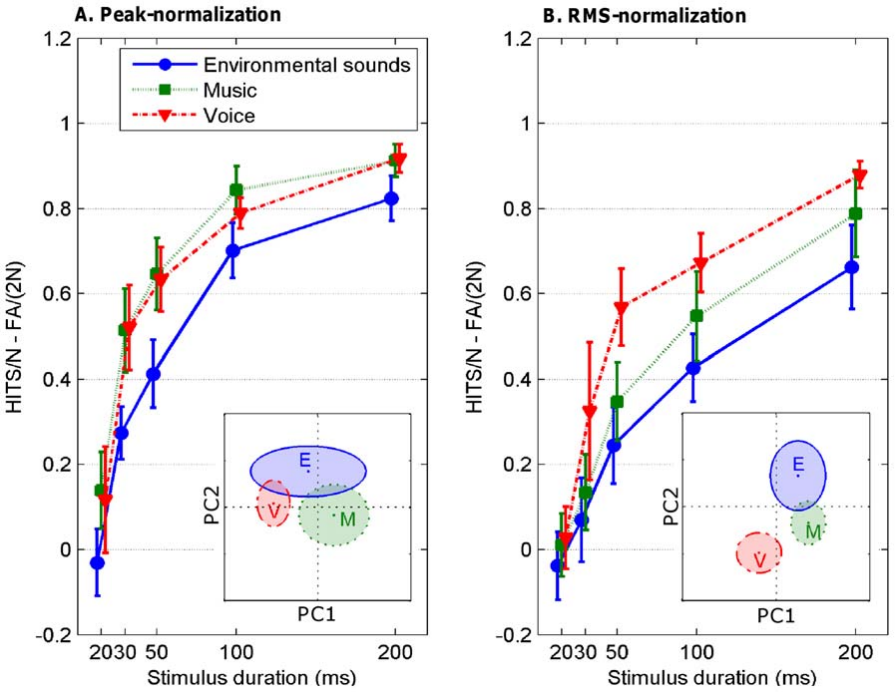
Participants' accuracy in the Peak-normalization (A) and RMS-normalized amplitude (B) conditions as a function of duration and sound category. Error bars represent the 95% confidence intervals of the mean. Inserts display the outcome of a PCA of the excitation patterns of all stimuli of all durations of Peak-normalization and RMS-normalization conditions. The center of each cluster indicates the barycenter within the PCA space, the horizontal and vertical lengths of the ellipses indicate the standard deviation of the items, on the first and second principal components, respectively. E refers to ESounds, M to musical sounds, V to voices, and PC to principal component. See Figure S1 for projection of the stimuli onto the PCA space as a function of stimulus duration.

All participants complained about the difficulty of the task, and most of them reported that voices were the most easily recognizable sounds. In order to analyze their performance, an index of accuracy [34] was defined by Hit/N – FA/(Nx2), with N being the number of items in each category for a given stimulus duration (N = 20 in this study). The accuracy for voice was thus: (Hits for voice)/20 – (FAs for voice)/40, the chance level being 0 (i.e., equal rates of Hits and FAs). The same calculation was applied for musical sounds and environmental sounds. As displayed in Figure 1, accuracy increased with duration, approaching the maximum value for 200 ms-stimuli. A 5 (Duration)×3 (Sound category)×2 (Normalization condition) ANOVA was performed with the first two factors as the within-subject variables, and the last one, as the between-subjects factor. Greenhouse-Geisser correction was used for the repeated measure analysis. Accuracy increased with duration, *F*(2.93, 102.38) = 260.46, MSE = 10.56, *p*<.0001. An increase of 10 ms in duration, between 20 ms and 30 ms, was sufficient to significantly improve performance for all sound categories, *F*(1, 35) = 84.97, MSE = 4.00, *p*<.0001. The other increments in duration had a more moderate impact, but all reached statistical significance (all *p*s<.0001). The main effect of sound category was also significant, *F*(1.69, 59.09) = 87.04, MSE = 1.77, *p*<.0001, with higher accuracy for music and voice stimuli than for ESounds, *F*(1, 35) = 274.18, MSE = 3.24, *p*<.01. This effect of sound category was more pronounced for some durations, as revealed by a significant interaction between sound category and duration, *F*(4.93, 172.30) = 3.05, MSE = 0.05, *p*<.02. There was also a main effect of normalization, with lower performance in the RMS-normalization condition, *F*(1, 35) = 20.17, MSE = 4.01, *p*<.0001. Interestingly, the three-way interaction was significant, *F*(4.92, 172.30) = 2.38, MSE = 0.04, *p*<.05: A voice superiority effect was found in the RMS-normalization condition only, with higher accuracy for voices than environmental and musical sounds, for durations longer than 20 ms (*F*(1, 35) = 90.88, MSE = 2.21, *p*<.001). In comparison to the Peak-normalization condition, the RMS-normalization reduced accuracy more strongly for environmental and musical sounds than for voices at durations greater than 20 ms, *F*(1, 35) = 18.29, MSE = 0.44, *p*<.001. For both normalization conditions, ESounds remained the most difficult sound source to recognize.

A striking finding was that voices and music were recognized above chance level at 20 ms (*t*(17) = 1.83, *p* = .08; *t*(17) = 3.02, *p*<.01, respectively) in the Peak-normalization condition, and at 30 ms in the RMS-normalization condition (*t*(18) = 3.9, *p*<.001; *t*(18) = 2.96, *p*<.01, for voice and music respectively). Recognition of ESounds was above chance level at 30 ms in the Peak-normalization condition, *t*(17) = 8.66, *p*<.0001, and 50 ms in the RMS-normalization condition, *t*(18) = 5.33, *p*<.0001.

Table 1 presents the percentages of labels chosen for each category of sounds, that is correct categorizations and mistaken categorizations (false alarms, FAs). We analyzed FAs given for each sound category with a 3×2 ANOVA (with Sound category as within-subject factor and Normalization condition as between-subjects factor). In addition to main effects of Sound category (F(1.58, 55.16) = 22.1, MSE = 1846.8, *p*<.001) and Normalization (F(1, 35) = 18.8, MSE = 3556.2, *p*<.001), this analysis confirmed an interaction between the Sound category and Normalization (F(1.58, 55.16) = 3.84, MSE = 321.2, *p*<.05). Even though RMS-normalization increased the number of FAs for all categories (in comparison to the Peak-normalization), the FA-rate for voice (35%) in the RMS-normalization condition was inferior to that for Esounds (48%) and music (41%), F(1, 35) = 19.82, MSE = 1618.5, *p*<.001 and F(1, 35) = 6.05, MSE = 284.6, *p*<.05, respectively. This analysis of correct categorization and mistaken categorization (False Alarms) suggests that the RMS-normalization of amplitude modulated the perceptual distance between the three categories, with RMS-normalization increasing the perceptual distance between voice and the two other categories.

**Table 1:** Percentages (%) of labels chosen for each category of sounds in the Peak-normalization (A) and RMS-normalization (B) conditions.

	Peak-normalization			RMS-normalization		
	Response			Response		
Stimulus	E	M	V	E	M	V
**E**	**60**	24	16	**52**	31	17
**M**	15	**77**	8	27	**59**	14
**V**	19	9	**72**	21	14	**65**

Bolds characters represent Hits (correct labels) and the other numbers represent False Alarms (errors, i.e., mistaken one category for another).

### Auditory modeling of the stimulus set

**Figure 2:**
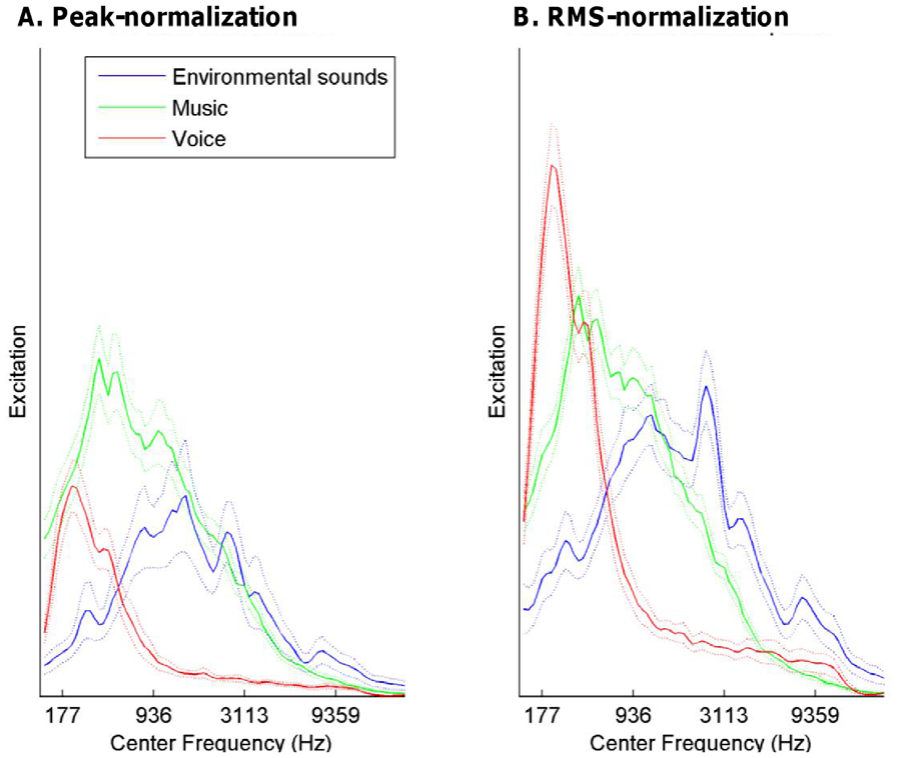
Average excitation patterns and 95% confidence intervals (indicated by dotted lines) for each set of stimuli, for the Peak-normalization (A) and RMS-normalized amplitude (B) conditions.

To further address this issue, auditory modeling of the set of stimuli was performed with a cochlear model [35], and a Principal Component Analysis (PCA) was run with the outcome of this analysis (performed on the entire set of stimuli). Specifically, the spectral envelope of each stimulus was split into 80 frequency bands using a gammatone filterbank [35] that simulates the frequency analysis performed by the cochlea. The RMS power was measured for each auditory filter, producing a so-called excitation pattern evoked by a given sound. Figure 2 shows the mean excitation patterns (and 95% confidence intervals) for each stimulus set average over all durations, for Peak-normalization (A) and RMS-normalization (B) conditions.

A global PCA of the excitation patterns of all stimuli of all durations of Peak-normalization and RMS-normalization conditions was run. The first two principal components accounted for 26% and 22% of the variance of the excitation patterns of the stimuli, and the remaining variance gradually decreased over the next components (i.e., 10%, 6%, 5%, 4%…). The obtained circle of correlation, which represents the projection of the original variables (i.e., the 80 frequency bands) on the PCA space, revealed that frequency bands centered at 1000 Hz and 247 Hz contributed the most to the principal components 1 and 2, respectively. As can been seen in Figure 2, these two frequency bands actually allow distinguishing the excitation patterns of the three sound categories, in the two normalization conditions. Hence, in the PCA space, the first two principal components allowed distinguishing the three types of sounds (Figure 1-left and 1-right, inserts). As illustrated by the size of the ellipses in Figure 1 (inserts), which indicates the standard deviations of the distribution for each sound category (on the first and second principal components, respectively), music and voice were found to have a lower spectral envelope variability compared to ESounds. Music and voice categories did not overlap. This can explain why sounds of both categories were weakly mistaken. However, music and voices overlapped with ESounds, for which they were mistaken (see Table 1). The outcome of the PCA changed depending on the normalization conditions in an interesting way. RMS-normalization increased the spectral distance between voice and ESounds, and had no effect on the distance between music and ESounds. The voice superiority effect observed in the RMS-normalization condition can thus be accounted for by an increased distance between voice and ESound categories and the removed overlap between these categories. The projection of the three sound categories on the global PCA space run separately for each condition of duration (see Figure S1) demonstrated that the difference in spectral variability of the three sound categories and their distance was weakly affected by the duration of the stimuli. This suggests that the stimuli contained enough acoustic information to potentially allow perceptual categorization, even at the shortest durations.

Multiple regression analyses were run to assess the influence of the spectral distributions and distances within the PCA space on response accuracy. The following predictors were entered in the regression models: 1) within-category distances (the average Euclidean distance of each item to the other members of its category), 2) between-category distances (average distance of each item of a given category to the members of the two other categories), and 3) the duration of the stimuli. In the Peak-normalization, we also entered the mean RMS power of each stimulus.

In the Peak-normalization condition, the four predictors accounted for 43% of the variance (adjusted R^2^ = .43, *F*(4, 595) = 112.34, *p*<.01, SSE = 21.79). Not surprisingly, there was a strong contribution of duration (β = .42, *p*<.01). Most interestingly, the within- and between- category distances contributed the most to the accuracy data (β = −.71, *p*<.01 and β = .32, *p*<.01, respectively): The smaller the distance with the actual category of the stimulus (within-category) and the bigger the distance with the stimuli of the other categories, the higher the accuracy. In addition, RMS amplitude provided a small contribution to the data (β = .26, *p*<.01): The louder the sounds, the higher the accuracy. In the RMS-normalization condition, the three predictors accounted for 48% of the variance in accuracy (adjusted R^2^ = .48, *F*(3, 596) = 182.37, *p*<.01, SSE = 18.03). There was again a strong contribution of the duration (β = .44, *p*<.01). The within-category and between-category distances contributed slightly, but significantly (β = −.10, *p*<.01 and β = .06, *p*<.05, respectively).

## Discussion

The present study demonstrates that sound categorization requires very little information. Up to now, this ability for processing reduced information of stimuli was mostly reported for the visual modality. The present findings are consistent with recent studies in auditory perception showing that less than 500 ms of sound is sufficient to recognize familiar tunes ([23]), to evaluate the emotion of music and even to identify the label of the pieces ([24], [36]). Our results went even one step further by showing that after 20 ms, sound categorization started to be above chance level, and accuracy was high for all sounds at 50 ms. This finding is striking given that the ecologically valid stimuli used in the experiment were cut at random places out of the original auditory signals. Moreover, none of the participants reported a specific expertise in auditory perception (e.g., music or sound engineering), and no training session was performed before the task. A further astonishing finding was that an increase of 10 ms in the auditory signal drastically boosts participants' performance.

The second contribution of the present study concerns the processes involved in the categorization of extremely brief stimuli. Neuroscience research has provided several arguments for human voice detectors, rooted in specialized neural pathways. Specialized detectors have several computational advantages compared to general systems. One of them is to respond in an automatic and fast way to stimuli they are specialized to detect. Processing advantages should thus be found for human voice processing, when compared to other auditory stimuli. Accordingly, better accuracy for voice stimuli was expected, notably at shortest durations. Most of the participants reported that voice stimuli were the sounds that were the easiest to-be-recognized. However, an advantage for voice stimuli was confirmed only in the RMS-normalization condition. Our study is the first one to document an effect of the RMS normalization procedure. Our data showed that RMS normalization had a strong detrimental effect on performance for all types of sounds, and modulated the way some categories of sounds can be recognized. In particular, the RMS normalization had a stronger detrimental effect for ESounds and music than for voice; and this led to a voice superiority effect only in the RMS-normalization condition.

It might be argued that the voice superiority effect observed in the RMS-normalization condition could be interpreted as an argument for voice-specialized pathways, as suggested by other studies. The fact that this finding was not replicated in the Peak-normalization condition could suggest that the lack of RMS normalization obscures the voice superiority effect. The mechanism that may “obscures” the voice superiority effect remains however unclear. If loudness were an important cue for perceptual categorization, the difference in loudness in stimuli should provide a strong contribution to participants' accuracy in the regression model. However, this was not the case. An alternative explanation is to consider that the processing advantage was only found in the RMS normalization condition, because it is a by-product of general categorization processes, which consider the distribution of the spectral envelope of the stimuli. This approach is more parsimonious as it does not require domain-specific processors, and it provides an alternative account of our entire data pattern. A critical finding along this line was the observation that the RMS normalization modified the distribution of spectral envelopes in a way that was significantly related to the changes in accuracy between the two normalizations conditions. As revealed by the regression analyses, the within-category distance provided the strongest contribution to human data - and in particular stronger than loudness (estimated by the average RMS energy) - in the Peak-normalization condition. Moreover, when the amplitude was RMS normalized, the within- and between-category distances contributed significantly to the data. This suggests that the distribution of perceptual features of the stimuli is a key determinant of auditory categorization, which can lead to a processing facilitation for voices in some loudness normalization conditions.

The present findings thus have methodological and theoretical implications. First, they reveal the necessity to control for the distribution of perceptual features of the stimulus set in order to be sure that a processing advantage for a given class of stimuli points to a specialized neural network. Given that most of the currently available studies did not provide this type of analysis, it is not possible to reject the more parsimonious interpretation that participants were responding on the basis of acoustic variability only. Second, given that the voice superiority effect has been reported with RMS-normalized sounds (e.g., [20], [27]), one might wonder about the adaptive advantage of this specialized network, if it turns out that this advantage is only observed for RMS-normalized stimuli. This advantage would thus be limited to material used in laboratory experiments. Our data do not deny the existence of specialized pathways for voice processing, but they point out that the processing advantage of this specific pathway for voice perception in everyday-life remains to be demonstrated.

## Supporting Information

Figure S1 **Projection of the experimental stimuli for the Peak-normalization (A) and RMS-normalized (B) conditions onto the PCA space, as a function of the stimulus duration.** The center of each cluster indicates the barycenter within the PCA space, the horizontal and vertical lengths of the ellipses indicate the standard deviation of the items, on the first and second principal components, respectively. E refers to ESounds, M to musical sounds and V to voices.
(TIF)

Table S1 **List of pieces from which musical sounds were extracted.**
(DOC)
