# Supplementary figures and images for: Categorization of Extremely Brief Auditory Stimuli: Domain-Specific or Domain-General Processes?
Source: PLoS One. 2011 Oct 27;6(10):e27024. doi: 10.1371/journal.pone.0027024 (PMC3203171; doi:10.1371/journal.pone.0027024)

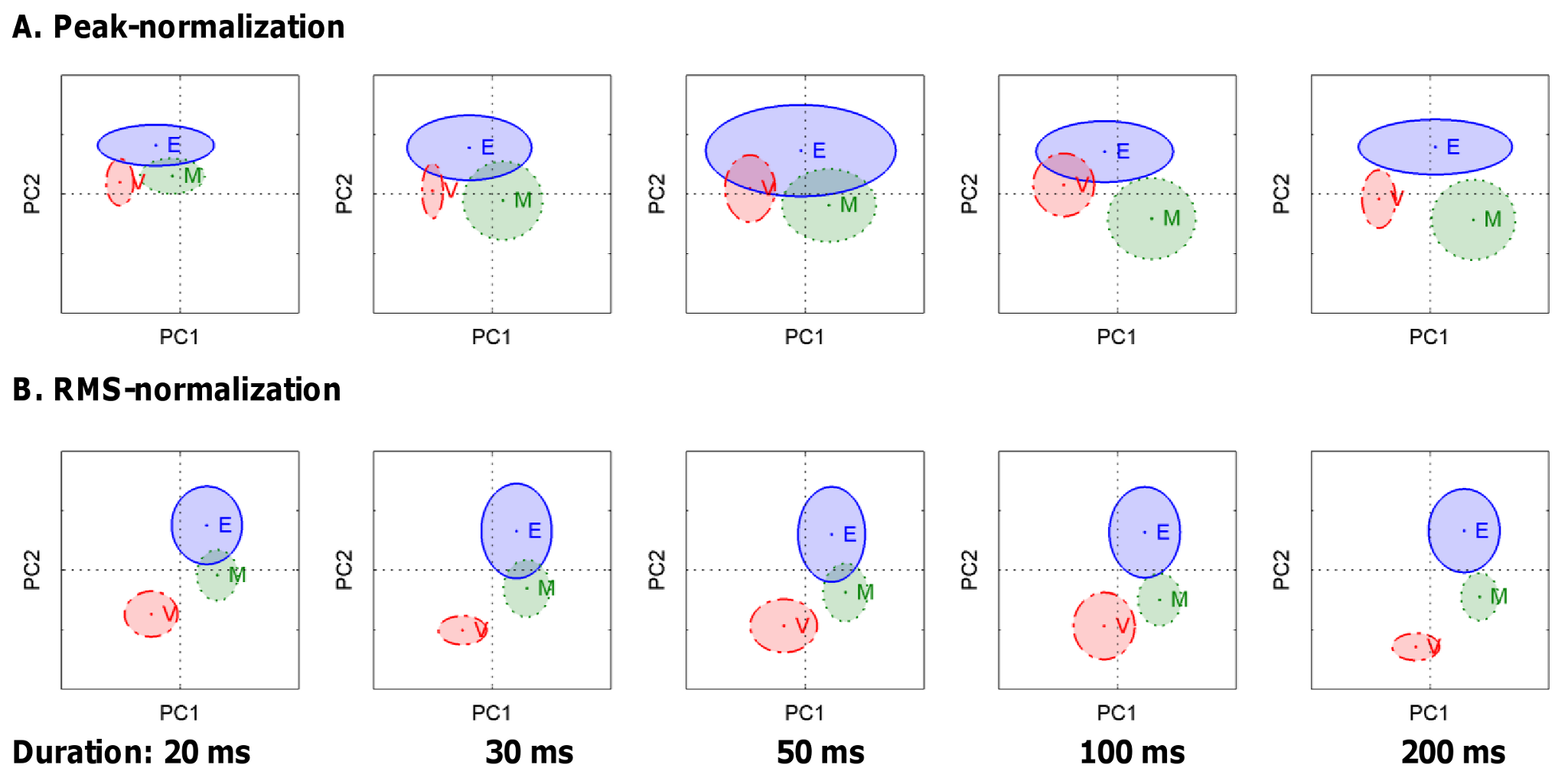

Supplement: Figure S1 — Projection of the experimental stimuli for the Peak-normalization (A) and RMS-normalized (B) conditions onto the PCA space, as a function of the stimulus duration. The center of each cluster indicates the barycenter within the PCA space, the horizontal and vertical lengths of the ellipses indicate the standard deviation of the items, on the first and second principal components, respectively. E refers to ESounds, M to musical sounds and V to voices. (TIF) [file pone.0027024.s001.tif]
