# Supplementary material for: Categorization of Extremely Brief Auditory Stimuli: Domain-Specific or Domain-General Processes?
Source: PLoS One. 2011 Oct 27;6(10):e27024. doi: 10.1371/journal.pone.0027024 (PMC3203171; doi:10.1371/journal.pone.0027024)
Supplement: Table S1 — List of pieces from which musical sounds were extracted. (DOC) [file pone.0027024.s002.doc]

| **Musical piece** | **Compositor** |
| --- | --- |
| Badinerie | Bach |
| Guillaume Tell - Overture | Rossini |
| Little night music | Mozart |
| Hungarian Dance No. 5 | Brahms |
| New world symphony, 4^th^ mvt | Dvorak |
| Boléro | Ravel |
| Jazz suite No. 2 | Shostakovitch |
| Carmen - Overture | Bizet |
| Symphony op. 11, 4th mvt | Olson |
| Symphony, VB 45 (Presto) | Kraus |
| I Vadstena Kloster, 3^rd^ mvt, Procession | Bengtsson |
| Symphony No. 1 - Final | Norman |
| Symphony No. 2 | Scriabine |
| Quintet for piano and winds, 2^nd^ mvt | Beethoven |
| Missommarvaka (rhapsodie) | Alfvén |
| The Valkyrie | Wagner |
| Wedding March | Mendelson |
| Sonata Doi Chori | Schmelzer |
| Drapa | Rubenson |
| Water music | Handel |
